# Strategies and Recent Advances in Tackling Antibacterial Resistance in India: A Comprehensive Narrative Review

**DOI:** 10.7759/cureus.95613

**Published:** 2025-10-28

**Authors:** Tanmay Goudar, Shreyas Desai, Manjunatha Basappa

**Affiliations:** 1 College of Medicine, Mysore Medical College and Research Institute, Mysore, IND; 2 Research, Owen J Roberts High School, Pottstown, USA; 3 Forensic Medicine and Toxicology, JSS Medical College, JSS Academy of Higher Education Research, Mysore, IND

**Keywords:** antibacterial resistance, antibiotics, factors influencing antibacterial resistance, india, self-medication, strategies to overcome antibacterial resistance

## Abstract

Antibacterial resistance (ABR) in India has emerged as a critical public health challenge. Increasing resistance to key antibiotics, including fluoroquinolones and carbapenems, in prevalent pathogens is driven by several factors. This review aims to summarize (1) the mechanisms of action of antibiotics and the emergence of resistance in India, (2) the multifactorial drivers of ABR across human, animal, and environmental sectors, and (3) public health strategic insights based on historical and contemporary literature to provide a roadmap for future interventions. A literature search was conducted using various keywords and databases such as PubMed, Scopus, and Google. Articles published between 1977 and 2025 were retrieved and reviewed. Additional data were integrated from national healthcare strategies and the Indian Priority Pathogens List. Over 200 articles were analyzed to understand the mechanisms of action of antibiotics, the evolution of resistance patterns, the underlying drivers, and the effectiveness of current policies and interventions.

The analysis revealed alarmingly high resistance rates, particularly among gram-negative bacteria such as *Escherichia coli*, *Klebsiella pneumoniae*, *Pseudomonas aeruginosa*, and *Acinetobacter baumannii*, as well as significant resistance among gram-positive pathogens such as methicillin-resistant *Staphylococcus aureus*. Key contributing factors include unregulated antibiotic sales, overprescription, inadequate infection control, and misuse in animal husbandry. The findings further underscore the benefits of integrated approaches, such as the One Health framework and targeted stewardship programs, although regulatory heterogeneity and limited surveillance infrastructure remain substantial obstacles. Addressing the ABR crisis in India requires an integrated, multi-pronged strategy. Over the next 5-10 years, India must prioritize developing and expanding robust surveillance systems for the timely detection of resistance trends, scaling up comprehensive antibiotic stewardship programs in clinical and community settings, and committing significant investment to research and development for new antibiotics and diagnostics. Embracing these forward-looking priorities is essential to mitigate ABR and secure sustainable healthcare outcomes.

## Introduction and background

Antibacterial resistance (ABR) is a critical global health challenge that arises when bacteria develop the ability to resist antibacterial drugs (antibiotics) that were once effective in treating infections [1-21]. ABR leads to treatment failures, persistent infections, and an increased risk of pathogen spread. Consequently, ABR leads contribute to higher rates of illness and mortality, along with escalating healthcare costs. 

India, as one of the world’s most populous countries, faces a particularly acute ABR crisis [22]. The country is at a pivotal juncture in its battle against ABR due to a complex interplay of factors, including prevalent self-medication practices, over-the-counter availability of antibiotics, widespread use of antibiotics in agriculture and animal husbandry, and insufficient healthcare infrastructure [23-26]. These challenges are compounded by socio-economic disparities, cultural practices, and environmental factors, creating hotspots for the emergence and spread of resistant pathogens [27-30].

The implications of ABR in India are profound, with ABR rates exceeding 70% for a class of antibiotics in pathogens like *Escherichia coli* and *Klebsiella pneumoniae* [31]. Resistance to last-resort antibiotics (e.g., carbapenems and colistin) is also alarmingly high, posing significant challenges to treatment [32-34]. The burden of drug-resistant tuberculosis (TB) further exacerbates the situation, with India bearing the highest global burden of multidrug-resistant TB (MDR-TB) and extensively drug-resistant TB (XDR-TB) [35-37].

Despite efforts to address ABR, including the development of the Indian Priority Pathogens List (IPPL) and the National Action Plan on Antimicrobial Resistance (NAP-AMR), significant gaps remain in surveillance, diagnostics, public awareness, and policy enforcement [38-42]. A comprehensive, multi-pronged approach is urgently needed to mitigate this public health threat.

This review addresses a critical gap in the current literature by providing an integrated, multifaceted analysis of ABR in India, an area where previous studies were predominantly siloed by discipline. Unlike earlier articles that focused largely on isolated aspects of resistance, this review synthesizes over significant studies to connect the dots between antibiotic mechanisms, local resistance patterns across key pathogens, and the socioeconomic, regulatory, environmental, and behavioral determinants driving the crisis. Furthermore, it introduces a comprehensive One Health framework that explicitly links human, animal, and environmental health sectors, thereby proposing novel, coordinated strategies for policymaking, antibiotic stewardship, and research and development. In doing so, the review not only highlights the evolving nature of resistance in pathogens such as fluoroquinolone- and carbapenem-resistant bacteria but also paves the way for future innovations in diagnostics and therapeutics tailored to India’s unique public health challenges. By addressing these gaps, the review aims to guide future policymaking, support new antibiotic research, and foster global collaboration in the fight against ABR.

## Review

Methodology

A non-formal, structured methodology was employed (note that adhering to PRISMA guidelines is not mandatory for writing a narrative review; see Table 1 for details). Articles published between 1977 and 2025 were retrieved from databases such as PubMed, Scopus, and Google Scholar. The search utilized specific keywords, including “antibacterial resistance in India,” “antibiotics in India,” “self-medication in India,” “factors influencing antibacterial resistance in India,” and “strategies to overcome antibacterial resistance in India.” Furthermore, cross-references from the retrieved articles were reviewed and incorporated into the analysis. Reports on the Indian Priority Pathogens List (IPPL), resistance rates, and national healthcare strategies were sourced from official government websites, including the Indian Council of Medical Research and the Department of Biotechnology, Government of India.

**Table 1:** Methodology details.

Aspect	Description
Inclusion Criteria	Peer-reviewed articles and reports that covered ABR in India, analyzed the mechanisms of antibiotics and their resistance, examined prescribing practices and antibiotic use in humans and animals, discussed environmental factors contributing to ABR, and proposed strategies to combat ABR, such as infection control, antibiotic stewardship, and public-private healthcare initiatives.
Exclusion Criteria	Articles were excluded if they lacked relevant information on ABR related to the Indian region, were published in languages other than English, or were not peer-reviewed or did not provide sufficient methodological rigor.
Data Extraction and Quality Assessment	Data extraction was conducted through one approach to ensure consistency, relevance, and accuracy. Key information was extracted from each study, including resistance rates and trends, mechanisms of antibiotics and their resistance, factors contributing to ABR (e.g., prescribing practices, socioeconomic factors and environmental influences), and proposed strategies to mitigate ABR. Quality assessment was performed to evaluate the methodological rigor of each study, focusing on aspects such as sample size, study design, data collection methods, and relevance to the research objectives. Studies that lacked sufficient methodological details, presented unclear findings, or did not meet the inclusion criteria were excluded from the synthesis. This approach ensured that only high-quality and relevant studies were included in the review, providing a robust foundation for evidence synthesis and actionable recommendations.
Evidence Synthesis	The synthesis of evidence involved analyzing the collected articles and reports to identify trends in resistance rates, mechanisms of resistance, and contributing factors. This process provided a comprehensive understanding of the prevalence of resistant pathogens, shedding light on the scope and scale of the ABR crisis in India. It also examined the mechanisms of action of antibiotics and the development of resistance, offering insights into how bacterial strains adapt and survive. Furthermore, the analysis explored the socioeconomic, cultural, and environmental factors driving the crisis, highlighting the interconnected nature of human behavior, healthcare practices, and environmental influences. Finally, the synthesis identified potential strategies to address ABR in India, including infection control measures, antibiotic stewardship programs, and public-private healthcare initiatives, providing actionable recommendations to mitigate the growing threat of ABR.
Discussion and Recommendations	The discussion section interpreted the findings from the evidence synthesis and provided actionable recommendations to address ABR in India. These recommendations were designed to tackle the multifaceted challenges posed by ABR and were grounded in the data analyzed. They emphasized the need for enhanced infection control measures, responsible antibiotic use, and the implementation of robust antibiotic stewardship programs. Additionally, the recommendations highlighted the importance of fostering public-private healthcare collaborations, improving diagnostic infrastructure, and promoting research into novel antibiotics and alternative therapies. By addressing the socioeconomic, cultural, and environmental factors driving ABR, these recommendations aimed to guide future research, policymaking, and healthcare practices, ensuring a comprehensive and sustainable approach to combating ABR in India.

Results

In this section, the mechanism of action of antibiotics and ABR, the ABR rate and trends, challenges in addressing the ABR, and potential strategies to address the ABR are summarized.

Mechanism of action of antibacterial drugs (antibiotics)

A summary of the primary mechanisms of action and examples of antibiotics is provided in Table 2 as a prelude to the following section on mechanisms of ABR in India [43-53]. Understanding the mechanisms of action of antibiotics is foundational for addressing resistance. This knowledge sets the stage for exploring how bacteria in India develop resistance (see next section below) and the challenges posed by resistant pathogens.

**Table 2:** Mechanism of action of traditional antibiotics and examples of antibiotics. The content included in this table represents the author's summary of the mechanisms of action based on published information.

Bactericidal Antibiotics: Antibiotics that kill bacteria directly by targeting essential bacterial processes	Mechanism of Action	Examples of Antibiotics
	Inhibition of bacterial cell wall synthesis: Disrupts peptidoglycan synthesis, leading to bacterial cell lysis.	Beta-lactams: Penicillins (e.g., amoxicillin), Cephalosporins (e.g., ceftriaxone), Carbapenems (e.g., meropenem), Monobactams (e.g., aztreonam); Glycopeptides: Vancomycin, Teicoplanin
	Disruption of cell membrane function/integrity: Alters membrane permeability, causing leakage of cellular contents.	Polymyxins: Colistin; Lipopeptides: Daptomycin
	Inhibition of nucleic acid synthesis: Blocks DNA or RNA replication, transcription, or repair.	Fluoroquinolones: Ciprofloxacin, Levofloxacin; Rifamycins: Rifampin, Rifabutin; Metronidazole
Bacteriostatic Antibiotics: Antibiotics that inhibit bacterial growth and reproduction	Inhibition of protein synthesis: Targets bacterial ribosomes, preventing protein synthesis.	Aminoglycosides: Gentamicin, Amikacin, Streptomycin; Tetracyclines: Doxycycline, Tetracycline; Macrolides: Erythromycin, Azithromycin, Clarithromycin; Lincosamides: Clindamycin; Chloramphenicol; Oxazolidinones: Linezolid
			Antimetabolite activity: Disrupts folic acid synthesis, essential for bacterial nucleotide production.	Sulfonamides: Sulfamethoxazole; Trimethoprim; Co-trimoxazole (Trimethoprim-Sulfamethoxazole)

Mechanisms of ABR in humans in India

The type of bacteria, the type of antibiotic resistant to the given bacteria, and the mechanism of resistance to a given antibiotic applicable to the Indian region are summarized in Table 3 [13,16,23,27,32,54-84]. 

**Table 3:** Bacteria found in India with antibiotic resistance, the type of antibiotics they are resistant to, and the mechanisms involved in their resistance. The content included in this table represents the author's summary based on published information.

Bacterium	Type of Antibiotic Resistant	Mechanism of Resistance
Escherichia coli	Carbapenems, Fluoroquinolones, Cephalosporins, Piperacillin-Tazobactam, Colistin	Beta-lactamase production: Enzyme that breaks down beta-lactam antibiotics. Efflux pumps and mutations: Increasing drug efflux and target site modification.
Klebsiella pneumoniae	Carbapenems, Cephalosporins, Piperacillin-Tazobactam, Colistin	Carbapenemase production (NDM, OXA-48): Enzymes that hydrolyze carbapenems. Efflux pumps.
Acinetobacter baumannii	Carbapenems, Cephalosporins, Colistin	Carbapenemase production: Enzymes including OXA groups. Efflux pumps and biofilm formation.
Pseudomonas aeruginosa	Carbapenems, Cephalosporins, Piperacillin-Tazobactam	Efflux pumps, Enzymatic degradation, Porin channel modification.
Staphylococcus aureus	Methicillin (MRSA), Vancomycin, Linezolid	mecA gene (MRSA), vanA/vanB genes (VRSA) leading to altered binding sites.
Enterococcus faecium	Vancomycin, Ampicillin	Modification of D-Ala-D-Ala to D-Ala-D-Lac (for vancomycin resistance). Beta-lactamase production (for ampicillin resistance).
Neisseria gonorrhoeae	Ciprofloxacin	Mutations in target genes (gyrA, parC) affecting drug binding.
Salmonella Typhi	Ciprofloxacin, Ceftriaxone, Ampicillin, TMP-SMX	Mutations in target genes (gyrA, parC), Efflux pumps, Decreased permeability.
*Mycobacterium tuberculosis *strains	Isoniazid	Mutations in katG gene (catalase-peroxidase enzyme)
	Rifampicin	Mutations in rpoB gene (RNA polymerase beta subunit)
	Ethambutol	Mutations in embB gene (arabinosyl transferase enzyme)
	Pyrazinamide	Mutations in pncA gene (pyrazinamidase/nicotinamidase enzyme)
	Streptomycin	Mutations in rrs gene (ribosomal RNA) and rpsL gene (ribosomal protein)
	Fluoroquinolones	Mutations in gyrA and gyrB genes (DNA gyrase subunits)
	Amikacin	Mutations in rrs gene (ribosomal RNA)
	Capreomycin	Mutations in tlyA gene (rRNA methyltransferase)
	Kanamycin	Mutations in rrs and eis genes (enhanced intracellular survival protein)

Indian Priority Pathogen List (IPPL) and its implications

The Indian Priority Pathogen List (IPPL) was developed by the World Health Organization’s Country Office for India in collaboration with the Department of Biotechnology, Government of India [85]. The primary objective of the IPPL is to identify the most critical antibiotic-resistant bacteria in India, strengthen infection control measures to mitigate resistance, and support the development of novel drugs and treatments [85]. Notably, Mycobacteria (including *Mycobacterium tuberculosis*) were excluded from this prioritization exercise, as they are already recognized as a global and national priority for which innovative treatments are urgently required and actively being developed [85]. A summary of the IPPL is provided in Table 4.

**Table 4:** Summary of Indian priority pathogens list. The content in this table represents a summary of the information provided in reference [85]. The priority classification is based on bacterial pathogens according to the species and resistance.

Priority Level	Description	Pathogen	Resistant to
Critical	This priority level includes pathogens with limited treatment options, high disease burden, and significant resistance trends.	Enterobacteriaceae	Carbapenem, tigecycline, and colistin
		Non-fermenting bacteria (e.g., *Acinetobacter baumannii* and *Pseudomonas aeruginosa*)	Carbapenem and colistin
High	This priority level includes pathogens with substantial disease burden and increasing resistance, but with more treatment options available.	*Staphylococcus aureus* (MRSA)	Methicillin, daptomycin and linezolid
		*Enterococcus* species	Vancomycin and linezolid
Medium	This priority level includes pathogens with moderate disease burden and resistance trends, but still requiring attention for public health measures.	Streptococcus pneumoniae	Cephalosporins and fluoroquinolones
		*Shigella* species	Cephalosporins and azithromycin

The development process of the IPPL involved a comprehensive literature review, data analysis of drug resistance mechanisms, expert consultation, and prioritization based on predefined criteria, including disease burden and resistance trends [85]. Data for the IPPL were sourced from systematic reviews, national antimicrobial resistance surveillance networks, and Indian biomedical literature databases [85]. Experts from diverse fields contributed to the prioritization process through a structured online survey [85].

The IPPL categorizes identified drug-resistant bacteria into three tiers: critical, high, and medium priorities (see Table 4). This tiered classification is essential for guiding focused antibiotic research and development investments towards the most impactful pathogens [86-90], thereby contributing to the effective management of the ABR crisis. Addressing the ABR crisis requires a thorough understanding of ABR rates and trends in India, as summarized below.

ABR rate and trends 

The key/prominent pathogens, the antibiotic classes to which they have developed resistance, and the reported approximate resistance rates across different regions of India are summarized in Table 5. This data is aggregated from various studies and the IPPL [13,16,23,27,32,54-60,85].

**Table 5:** Regional distribution of ABR in India. Notes: Some studies also report declining resistance for older antibiotics (e.g., ampicillin and TMP-SMX in *Salmonella Typhi*) [13,16,23,27]. The numbers above represent a synthesis from several reports; details (e.g., exact resistance rates) can vary by specific hospital and study period.

Region	Pathogen	Antibiotic Class/Agent	Approximate Resistance Rate	Notes	References
Northern India	*Staphylococcus aureus* (MRSA)	Methicillin	~47%	Data reported from tertiary centers in North India; similar trends noted nationwide.	[13,16,23,27,32,54-60]
Eastern India	Escherichia coli	3rd-Generation Cephalosporins	>70%	Studies from referral hospitals in Eastern India show high cephalosporin resistance.	[13,16,23,27,32,54]
Southern India	Pseudomonas aeruginosa	Carbapenems	40–50%	Surveillance data from Southern tertiary care hospitals indicate rising carbapenem resistance.	[13,16,23,27,32,55-60]
Western India	Acinetobacter baumannii	Fluoroquinolones / Carbapenems	>70%	Data from regional monitoring networks and the IPPL reveal very high resistance rates.	[13,16,23,27,32,54-60, 85]
Nationwide (Various Regions)	Klebsiella pneumoniae	Carbapenems	~57%	National surveillance indicates heterogeneous but consistently high carbapenem resistance across regions.	[13,16,23,27,32,54-60]

ABR trends over time for key or prominent pathogen-antibiotic combinations are presented (Table 6). This data is based on longitudinal surveillance reported in previous studies [13,16,23,27,32,54-60].

**Table 6:** Temporal trends in ABR rates in India. *Notes: Exact numbers may vary based on the study design and local factors; these ranges represent typical findings in multicentric studies. Data sources include large private laboratory networks and national surveillance reports [54,85].

Pathogen	Antibiotic/ Antibiotic Class	Earlier Period (Approximate)	Recent Period (Approximate)	Notes	Reference
E. coli	Fluoroquinolones	~65% (2008–2014)*	>70% (2015–2021)*	Steady increase noted; resistance now exceeds 70% in many settings.	[54,13,16]
Klebsiella pneumoniae	Carbapenems	~40–45% (prior to 2015)	~57% (2015–2021)	Significant rise in carbapenem resistance over recent years.	[13,54,55,56]
Pseudomonas aeruginosa	Third-generation cephalosporins/ Carbapenems	~45% (2008–2014)*	~40–50% (2015–2021)*	Resistance rates remain high with a slight upward trend overall.	[13,16,23,27,32,54-60]
Acinetobacter baumannii	Fluoroquinolones / Carbapenems	>70%	Remain >70%	Resistance rates are persistently high with limited improvement over time; often exceeding 70%.	[13,16,23,27,32,54-60]

In summary, Tables 5, 6 present an overview of regional variations and temporal trends in ABR in India. Regarding regional differences, Western India frequently reports the highest resistance levels, with rates exceeding 70% for certain pathogens and antibiotics. However, Northern, Eastern, and Southern regions also show alarmingly high resistance rates across multiple pathogens. The data further highlight increasing resistance rates over time, particularly among key multidrug-resistant bacteria such as *Escherichia coli* (fluoroquinolones) and *Klebsiella pneumoniae* (carbapenems).

Multifaceted and interconnected challenges in combating ABR in India

The challenges of combating ABR in India reveal the interplay of socio-economic, environmental, regulatory, and healthcare factors [1-204]. Addressing these challenges necessitates a deeper understanding of the burden of bacterial infectious diseases and their impact on public health.

Burden of bacterial infectious diseases

Bacterial infections such as tuberculosis (TB), typhoid fever, bacterial pneumonia, and diarrheal diseases continue to pose a significant public health burden in India [1,3,12,19,23,30,65-84]. TB remains a particularly critical concern, with India accounting for the highest number of cases globally [65-84]. These infections result in substantial mortality and place considerable economic strain on healthcare systems and affected families.

Several interconnected factors, as summarized below, contribute to this ongoing burden. These include high population density, diverse socio-economic conditions, antibiotic resistance, and limitations in public health infrastructure [1-110].

The pervasive impact of bacterial infections highlights the urgent need for immediate and sustained strategies to reduce infection rates and improve treatment outcomes. Addressing this issue requires curbing the improper use of antibiotics and strengthening healthcare practices to ensure better prevention, diagnosis, and care.

Improper use of antibiotics

The improper use of antibiotics manifests in various ways, including over-prescription by healthcare providers, self-medication, non-compliance with prescribed regimens, and the sale of antibiotics without prescriptions [15,111-124]. These practices contribute to the growing challenge of antibiotic resistance, which undermines the effectiveness of treatments for bacterial infections and poses a serious threat to individual and public health.

One major driver of antibiotic misuse in India is the over-prescription of these drugs by healthcare providers [114-116,119,121,124]. Factors such as limited access to diagnostic facilities, pressure from patients seeking quick remedies, and insufficient training in antimicrobial stewardship play a significant role in this trend [114-124]. In many cases, antibiotics are prescribed for viral infections, despite their ineffectiveness against viruses [116,124-128]. This not only leads to unnecessary antibiotic use but also accelerates the development of antibiotic resistance in bacterial populations.

Even when antibiotics are prescribed correctly, non-compliance among patients remains a widespread issue. Some patients prematurely discontinue their medication upon experiencing symptom relief, while others may stop due to financial constraints [122-124]. Incomplete courses of treatment fail to fully eradicate bacterial infections, increasing the risk of resistant strains emerging.

The improper use of antibiotics underscores the urgent need for stricter regulations and enhanced public awareness. Addressing these challenges requires exploring regulatory and policy solutions to effectively combat antibiotic resistance.

Regulatory and policy challenges

The lack of uniform, stringent regulations regarding antibiotic use, as well as inconsistent implementation across states, complicates efforts to restrict over-the-counter sales and enforce prescription-only policies [7,24,29,30,32,88,109,120,129-131,153,154,176]. 

Fragmented policies and decentralized enforcement hinder the nationwide management of ABR. Regulatory bodies like the Food Safety and Standards Authority of India (FSSAI) and the Central Drugs Standard Control Organization (CDSCO) face resource constraints and lack coordination, undermining their efficacy.

Regulatory and policy challenges emphasize the need for coordinated action between Indian and international bodies and enforcement to curb antibiotic misuse. Addressing these challenges requires understanding the socio-economic and behavioral factors influencing antibiotic use.

Socioeconomic and behavioral factors

India's socioeconomic and behavioral diversity leads to varied health practices, making the containment of ABR challenging [32,92,118,122,124,132,133]. Economic factors, such as poverty, play a significant role in determining access to proper healthcare and influence how antibiotics are used. Financial constraints often compel individuals to seek cheaper, over-the-counter alternatives rather than consulting qualified healthcare professionals. Addressing these socioeconomic and behavioral challenges requires targeted interventions. These include increasing public awareness, improving healthcare access, and implementing stricter regulations on the sale of antibiotics.

Socioeconomic and behavioral factors also intersect with environmental drivers of antibiotic resistance, emphasizing the need for a holistic, multidisciplinary approach to tackle this issue effectively.

Environmental factors

Environmental factors significantly contribute to the emergence and spread of resistant bacteria [83,136,139,115,136,139,143]. Pharmaceutical manufacturing facilities in India sometimes release antibiotic residues into the environment through untreated wastewater, contaminating water bodies, soil, and crops. Hospital and agricultural waste containing antibiotics and resistant bacteria often goes untreated, leading to environmental dissemination [138,140,141]. Urban areas face challenges related to overcrowding, poor sanitation, and limited access to clean water [14,32,83,115,142,143], amplifying the risk of resistant pathogens spreading. Antibiotics are extensively used in animal husbandry and aquaculture, fostering resistant strains that can transfer to humans through the food chain.

Addressing the environmental factors requires long-term based and sustainable practices. For example, enhancing the nation's effort in antibiotic research and development to combat ABR effectively.

Research and development constraints

The research and development (R&D) of new antibiotics to combat ABR in India is faced with multidimensional challenges, stemming from scientific, economic, policy, and societal issues [18,27,104,109,144,152,179].

Finding new antibiotics is a complex task, as it requires identifying molecules that can effectively target bacterial pathogens without harming human cells [5,6,135,144-147]. The mechanism of ABR is continually evolving, making the research landscape a moving target [5,6,135,145-147]. Despite advances in microbiology and genomics, there is still a limited understanding of the natural environments where potential antibiotic compounds might be found, contributing to the slow pace of discovery [24,148].

Developing new antibiotics involves expensive, lengthy, and high-risk processes [23,29,87,144,149]. The market return for antibiotics is typically low compared to chronic disease drugs due to the short-course nature of antibiotic treatments [23,29,149,150]. In India, limited financial incentives and support for pharmaceutical companies deter significant investment in antibiotic R&D [150-152]. The industry faces challenges such as inadequate patent protection and price caps, which further reduce profitability and discourage innovation [15,153,154].

Complex regulatory requirements and bureaucratic hurdles slow down the approval process for new antibiotics [15,87,144,149,151]. While India has initiated programs to address ABR, such as the National Action Plan [88,130], effective implementation is critical for the rapid development of novel antibiotics.

The constraints in R&D underscore the need for investment and innovation to develop novel antibiotics and alternative therapies. These efforts must be complemented by public awareness and education initiatives.

Public awareness and education

In India, the lack of public awareness and education about the proper use of antibiotics and the serious consequences of their misuse is a major socio-economic and cultural driver [88,130]. A prevalent misconception among the Indian population is the belief that antibiotics are universal remedies for a wide range of illnesses, including viral infections such as the common cold and flu, conditions for which antibiotics are entirely ineffective [88,130,125-127,155-157]. 

Additionally, healthcare providers, particularly those in rural areas and small clinics, often face challenges stemming from inadequate training in antimicrobial stewardship [113,114,119]. This lack of training can result in improper prescribing practices, such as the routine prescription of antibiotics for viral infections or yielding to patient demands for stronger medications.

Improving public awareness and education is crucial for fostering responsible antibiotic use and combating resistance. These efforts must be integrated into multi-pronged strategies to address ABR comprehensively. 

Variability in ABR surveillance in India

The surveillance of ABR in India reveals several differences and limitations that must be considered when interpreting the findings of this review and formulating strategic decisions. Key areas of variability in surveillance data and methodological challenges in Indian studies related to antibiotics are summarized below:

Regional Variability

Significant differences in antimicrobial susceptibility patterns across regional centers were observed [90]. This variability underscores the importance of developing localized strategies to combat antimicrobial resistance, rather than relying on generalized data that may not accurately represent regional nuances.

Community vs. Hospital Data

Most data were collected from tertiary care hospitals and do not reflect community-level antimicrobial resistance patterns [90]. Caution should be exercised when extrapolating hospital-based findings to broader community settings.

Impact of COVID-19

The misuse and overuse of antibiotics during the COVID-19 pandemic have contributed to declining susceptibility rates, particularly for carbapenems and colistin [90]. This trend underscores the need for heightened awareness and regulation of antibiotic use during public health crises.

Genotypic vs. Phenotypic Resistance

Not all isolates demonstrated a consistent correlation between genetic mutations and phenotypic resistance [90]. This finding highlights the complexity of resistance mechanisms, which are influenced by factors such as gene expression and antibiotic selection pressure.

Scope of Data Collection

While the report analyzed 99,492 culture-positive isolates, its focus on six key pathogenic groups may limit insights into other emerging pathogens or resistance trends [90]. A broader scope of data collection is necessary to capture a more comprehensive picture of ABR.

Sample Bias

The data were predominantly obtained from ICU, ward, and outpatient department (OPD) settings [90], which may not adequately represent the broader population or community-level resistance patterns. This sample bias highlights the need for more inclusive data collection methodologies.

These findings emphasize the importance of refining surveillance methodologies, expanding the scope of data collection, and implementing localized interventions to effectively address the challenges posed by antimicrobial resistance in India. A more tailored approach is critical to developing actionable strategies that account for regional, community, and pathogen-specific variability.

Multi-pronged approaches to combat ABR in India

Isolated interventions targeting a single aspect of ABR are insufficient for sustainable outcomes. A multi-pronged, coordinated approach that integrates clinical, environmental, and societal strategies is essential [31,33,36,37,52,82,89,104,105,158,161]. This approach enables long-term results, reduces the risk of resistance recurrence, and promotes adaptability to emerging threats, such as new resistance mechanisms or outbreaks of resistant infections [2,43,52,101]. This strategy highlights the importance of improving infrastructure for R&D and diagnostics.

Strengthen R&D and diagnostic infrastructure

Advancing R&D capabilities are pivotal for developing novel antibiotics, alternative therapies, and innovative tools to address ABR effectively [37,87,134,147,162-164]. However, challenges such as inadequate funding, limited access to advanced technologies, and shortages of skilled personnel hinder progress [18,82,93,107,151,165]. Key priorities include increasing R&D investment, establishing specialized research centers, fostering collaborative networks, and incentivizing innovation through grants, tax benefits, and awards [15,30,93,130,151-152,158,165].

Public-private partnerships can provide sustained financial support for antibiotic development and diagnostic research. Dedicated research hubs should focus on resistance mechanisms, new drug targets, and improving existing antibiotics. International collaborations with organizations such as the WHO’s Global Antimicrobial Resistance Surveillance System can provide access to advanced technologies and expertise [33].

Accurate diagnostics are equally vital for combating ABR. Establishing affordable, accessible diagnostic laboratories and promoting point-of-care (POC) diagnostics can enable rapid identification of resistant infections and facilitate targeted treatments. Integrating diagnostic tools into primary healthcare systems and training healthcare workers in their use can improve antimicrobial stewardship at the grassroots level [3,29,38,90,157]. Strengthening surveillance systems and standardized reporting will guide evidence-based public health interventions [54,56].

A coordinated effort among government agencies, healthcare institutions, and international organizations is necessary to prioritize ABR as a public health emergency. Public awareness campaigns emphasizing the risks of antibiotic misuse and the importance of diagnostics are also critical for fostering evidence-based practices [23,24,82,112,156].

Develop alternative treatment strategies

Innovative approaches targeting bacterial vulnerabilities are emerging as promising solutions to combat ABR (Table 7). These include leveraging antimicrobial peptides (AMPs), artificial intelligence (AI), and other advanced technologies [37,40,44,135,162-164]. By broadening the scope of antibacterial options, alternative strategies complement conventional methods and lay the foundation for sustainable, long-term solutions.

**Table 7:** Alternative therapeutic or new antibiotic development strategies, their mechanisms, and their relative differences compared to traditional antibiotics. The content included in this table represents the author's summary based on published information.

Alternative Strategy	Description	Mechanism	Advantages Over Traditional Antibiotics
Antimicrobial peptides (AMPs)	AMPs are naturally occurring short peptides that play a role in the innate immune response in various organisms, including humans, plants, and animals. The AMPs exhibit broad-spectrum antimicrobial activity against bacteria, fungi, and viruses.	AMPs disrupt microbial cell membranes through electrostatic interactions that destabilize the lipid bilayer, leading to cell lysis. Some AMPs can penetrate cells and interfere with intracellular targets. They can also modulate immune responses and disrupt biofilms (complex communities of bacteria).	Compared to traditional antibiotics, AMPs have a broader spectrum of activity and are less likely to lead to resistance due to their multifaceted mechanism of action. They can be used alone or in synergy with antibiotics.
Antibiotic-peptide conjugates	This strategy involves chemically linking traditional antibiotics with AMPs (antibiotic-peptide conjugates) to enhance stability, specificity, and the antimicrobial spectrum.	Antibiotic-peptide conjugates can enhance cellular uptake, inhibit efflux pumps, and disrupt cellular processes both extracellularly and intracellularly.	Compared to traditional antibiotics, antibiotic-peptide conjugates offer increased specificity, stability, and efficacy, especially against resistant strains. They provide a dual mode of action (dual targeting) compared to monotherapy with traditional antibiotics, which is particularly useful against resistant strains.
Reintroduction of forgotten antibiotics	This strategy includes re-using older antibiotics such as colistin and fosfomycin for their effectiveness against modern pathogens, especially MDR strains.	These antibiotics typically interfere with cell wall synthesis or metabolic processes crucial for bacteria.	Cost-effective options with a history of proven efficacy; limited regulatory hurdles since they’re already approved, and they can bypass the extensive R&D process required for new drugs.
				Combination therapies	This strategy includes combining antibiotics with non-antibiotic compounds such as AMPs, nanoparticles, peptides, biofilm inhibitors, and metabolic disruptors for enhanced efficacy and to overcome resistance.	This approach targets multiple bacterial pathways, disrupts biofilms, enhances drug uptake, and leverages synergy to improve outcomes.	Synergistic effects can reduce the necessary dosages, minimize toxicity, and decrease the evolution of resistance mechanisms due to multifaceted targeting.
Vaccines and phages	Vaccines are being developed to prevent bacterial infections, thereby reducing the need for antibiotics. Bacteriophages (phages) specifically target and kill bacteria.	Vaccines boost the immune system to fight specific bacterial infections. Phages penetrate bacterial cells and lyse them, reducing bacterial load.	Long-term prevention strategies (vaccines) reduce the incidence of infection, while phages offer a highly specific approach with less likelihood of resistance development.				
Artificial intelligence (AI)	AI and machine learning are being utilized to design novel AMPs and identify new compounds by analyzing large datasets and predicting antimicrobial properties, enhancing the efficiency and success rate of drug discovery.	AI algorithms can quickly process data to predict peptide sequences with antimicrobial activity, optimizing drug discovery.	Increased efficiency in drug design and discovery, reduction in time and costs associated with traditional experimental approaches.
Exploration of new habitats and cultivation techniques	Researchers are exploring previously untapped environments such as marine settings and deploying innovative cultivation methods like co-cultivation or iChip to discover novel antibiotic producers.	Utilization of innovative technologies such as microfluidics, iChip devices, and co-cultivation approaches can isolate new microorganisms and discover unique bioactive compounds.	These approaches have the potential to uncover entirely new classes of antibiotics with unique structures and mechanisms, potentially avoiding existing resistance pathways.
				Antisense oligonucleotides (ASOs)	ASOs are short, synthetic sequences of nucleotides designed to bind specifically to bacterial RNA, inhibiting translation and reducing expression of target genes involved in resistance.	ASOs work by hybridizing to complementary mRNA sequences, preventing translation and leading to mRNA degradation. This specific targeting can potentially silence genes responsible for antibiotic resistance.	Offers precise targeting of bacterial genes without affecting non-target organisms, thus minimizing off-target effects and resistance development.
Macrocycles and cyclic peptides	Macrocycles are large, cyclic molecules that can disrupt bacterial cell processes more effectively than many linear small molecules due to their unique structural properties.	These compounds can target proteins and pathways distinct from those targeted by traditional antibiotics, offering new approaches to combat resistant bacteria.	Structural diversity leads to varied mechanisms of action compared to traditional antibiotics, potentially bypassing existing resistance mechanisms.				
Genomic and proteomic analysis for target identification	Deep genomic and proteomic profiling enables the identification of novel bacterial targets for therapeutic intervention, potentially leading to new classes of antibiotics.	By analyzing bacterial genomes and proteomes, researchers can identify unique, previously unexploited targets for new antimicrobial agents.	Identifies bacterial vulnerabilities that are not addressed by existing antibiotics, paving the way for novel treatment avenues.

Successful implementation requires collaboration among researchers, clinicians, and policymakers to ensure accessibility and integration within healthcare systems. These strategies align with the One Health framework, which integrates human, animal, and environmental health to address interconnected drivers of ABR.

Integrate a One Health strategy across India

The One Health approach in India holds significant promise by integrating human, animal, and environmental health strategies to combat ABR [30,36,89,158,172,174,175,181,191]. However, its implementation has been hindered by several critical barriers that require careful attention. One major challenge arises from the fragmented governance and regulatory oversight inherent in India’s diverse healthcare landscape. Despite well-conceptualized policies, the decentralized structure of the health system has led to weak intersectoral coordination and variable enforcement of regulations. Regulatory bodies such as the Central Drugs Standard Control Organization (CDSCO) and the Food Safety and Standards Authority of India (FSSAI) often lack sufficient resources, resulting in inconsistent application of prescription-only sales and antimicrobial stewardship initiatives across different regions [30,32,88,130]. This issue is compounded by the limited infrastructure and capacity deficits, particularly in rural areas where diagnostic laboratories and trained personnel are scarce.

The rural-urban divide further complicates the effective execution of the One Health strategy. Urban centers generally benefit from better-equipped hospitals and more robust infection control practices, whereas rural regions often suffer from sparse diagnostic facilities and inadequate ABR surveillance systems [30,36,89,158,172,181]. This disparity affects not only the accessibility of healthcare services but also the level of awareness regarding proper antibiotic use. The uneven distribution of resources has led to a higher propensity for self-medication and over-the-counter antibiotic sales in rural areas, creating significant surveillance blind spots that undermine national efforts [8,14,28,32,92]. In addition, differences between public and private sector practices exacerbate the situation; while public hospitals tend to follow standardized antimicrobial stewardship protocols, many private facilities, ranging from well-equipped tertiary centers to small, unregulated clinics, often lack uniform guidelines. Economic incentives in these private settings may even promote the liberal use of broad-spectrum or last-resort antibiotics, further contributing to the rise in resistance [121,154].

The COVID‑19 pandemic has further illuminated existing vulnerabilities within India’s ABR management framework. During the pandemic, increased reliance on empirical antibiotic treatments, driven by diagnostic uncertainties, appeared to fuel the development of resistance, as clinicians frequently resorted to broad‑spectrum antibiotics in the absence of rapid and accurate diagnostic tools [168,205-207]. Meanwhile, the diversion of healthcare resources toward COVID‑19 response efforts disrupted routine ABR surveillance, particularly in rural and resource‑constrained settings, leading to significant data gaps and delayed corrective measures [168,205-207]. These experiences underscore the necessity of establishing resilient, integrated surveillance systems capable of operating effectively during public health emergencies, ensuring that ABR monitoring remains uninterrupted despite competing healthcare priorities.

To address these multifaceted challenges, stakeholders must adopt evidence-based, granular recommendations that target the core issues hindering the successful integration of One Health strategies in India (see an example in Table 8). First, strengthening intersectoral coordination is essential. This can be achieved by establishing a centralized, interoperable data platform that aggregates surveillance data from human, animal, and environmental sources, and by forming permanent One Health committees at both the central and state levels to ensure collaborative policy implementation. Second, enhancing rural capacity through investments in affordable point-of-care diagnostic tools and robust laboratory infrastructure is critical. Comprehensive training programs for rural healthcare workers must also be established to curtail self-medication practices and improve targeted antibiotic use. Third, there is an urgent need to harmonize practices between the public and private sectors. Mandating standardized antibiotic prescribing protocols across all healthcare facilities and offering incentives, whether financial or technological, to private providers can foster uniformity in antimicrobial stewardship efforts. Lastly, leveraging lessons learned during the COVID-19 pandemic is vital. Building resilient surveillance systems that can withstand public health emergencies and incorporating digital health solutions and telemedicine platforms to expand the reach of education and stewardship programs have proven effective during the pandemic and should be integrated into long-term strategies.

**Table 8:** One Health-based strategies to combat ABR (strategy areas, target sectors and key actions that need to be taken to implement the strategy in target sectors). The content included in this table represents the author's summary based on published information.

Strategy Area	Target Sectors	Key Actions Necessary for Implementation
Global collaboration	Human, animal, environment	The World Health Organization (WHO), Food and Agriculture Organization (FAO), World Organization for Animal Health (OIE), and the United Nations Environment Programme (UNEP) have established a joint action plan under the one health approach. This plan emphasizes a holistic approach that integrates human, animal, plant, and environmental health. WHO’s strategic frameworks advocate collaborative action across sectors to reduce inappropriate antimicrobial use and enhance surveillance and monitoring.
Awareness and education	Public, healthcare workers	World Antimicrobial Awareness Week (WAAW) is an annual campaign to raise awareness about ABR and promote responsible antimicrobial use across public and healthcare sectors globally.
		Implement educational initiatives for farmers, healthcare providers, and community members to encourage responsible use and reduce antibiotic overuse.
Surveillance and technology	Human, animal, environment	Establish systems that monitor antibiotic use and resistance patterns in human, animal, and environmental health. Incorporate molecular tools, such as Whole Genome Sequencing and metagenomics, to understand the genetic basis of resistance and track its spread.
Regulation and policy	Human, animal health	India has banned specific antimicrobials, such as colistin, in food animal production. Regulatory frameworks like the National Action Plans on ABR aim to control antibiotic misuse. Develop National Action Plans (NAPs) aligned with WHO’s Global Action Plan to combat ABR by improving surveillance, promoting responsible use, and encouraging multisectoral collaboration.
Research and innovation	Human, animal, environment	Encourage investments in the development of new antimicrobials and alternative therapies. Focus on innovative solutions that address ABR in humans, animals, and the environment. Create organizations such as Indian Network for Fisheries and Animal Antimicrobial Resistance to facilitate research and policy implementation through one health perspectives.
Environment and hygiene	Human, environmental	Ensure proper disposal of pharmaceutical and agricultural waste to minimize environmental contamination and the spread of resistant bacteria. Implement safe practices in healthcare settings, farming, and food production to reduce ABR and ensure better health outcomes.

By critically engaging with these barriers, including fragmented governance, rural-urban disparities, and the disruptive impacts of COVID-19, and by learning from previous underperforming interventions, this deeper analysis provides actionable, evidence-based recommendations. These strategies address systemic challenges and underscore the importance of tailoring interventions to India’s diverse socio-economic contexts, ultimately paving the way for a more resilient and effective One Health approach in combating antibacterial resistance.

Effective implementation of One Health frameworks requires robust government policies and management to ensure sustainable progress.

Implement and manage government policies effectively

Recognizing the urgent need to address ABR, the Indian government launched the National Action Plan on Antimicrobial Resistance in 2017, aligned with the WHO’s Global Action Plan on ABR [32,88,130,174,175]. This policy framework focuses on three critical areas. First, raising public awareness is foundational; education campaigns target healthcare providers and communities to promote responsible antibiotic use and highlight the dangers of misuse [30,88,130,174,175]. Second, strengthening surveillance systems is crucial; the Indian Council of Medical Research supports the development of laboratory networks across the country to track resistance patterns and inform evidence-based policies [30,88,130,175]. Third, regulatory measures such as amendments to the Drugs and Cosmetics Rules (e.g., Schedule H1) mandate prescriptions for specific antibiotics to curb over-the-counter sales and reduce inappropriate usage [154,176,177]. These policy efforts aim to address the root causes of ABR and improve antibiotic stewardship.

Despite the comprehensive design of these policies, India faces significant barriers to their effective implementation. Financial constraints often limit the scalability of initiatives like surveillance networks, public education campaigns, and research into ABR solutions [36,42,97,105,109,138,149,157,178]. Additionally, rural areas face critical gaps in healthcare delivery, laboratory capacity, and diagnostic infrastructure, making it difficult to ensure consistent enforcement of ABR measures across the country [18,36,56,105,157,159,168]. Fragmented coordination among key sectors, including health, agriculture, and education, also hampers policy execution [30,36,88,89,117,130,158,172]. A multisectoral approach is essential to address the interconnected drivers of ABR, yet collaboration among stakeholders remains disjointed, creating inefficiencies.

To overcome these challenges, India requires a proactive and dynamic strategy. Public-private partnerships can play an instrumental role in mobilizing resources, fostering innovation, and leveraging expertise from diverse stakeholders such as NGOs, academia, and private healthcare facilities [21,36,82,83,107,158,179-181]. These efforts must be supported by antibiotic stewardship programs to optimize antibiotic use.

Embrace and implement the antibiotic stewardship program effectively

The concept of antibiotic stewardship is increasingly becoming integral to global healthcare. This program consists of coordinated strategies aimed at the responsible use of antibiotics, improving healthcare practices, and fostering collaboration between relevant stakeholders to enhance patient outcomes, reduce the development of antibiotic resistance (ABR), and preserve the effectiveness of existing antibiotics for future generations.

It is crucial for Indian healthcare institutions and related stakeholders to adopt and implement strategies such as hospital-based stewardship programs, community-level initiatives, capacity building, integration of advanced technologies, policy and regulation, and infection prevention and control to combat and stay ahead of ABR as summarized below. These initiatives must be complemented by infection prevention and control measures to minimize the spread of resistant pathogens.

Hospital-based stewardship programs

Hospitals are often the primary sites for antibiotic prescriptions and locations where resistant infections are most prevalent. Therefore, they play a critical role in addressing ABR issues. Multidisciplinary stewardship teams, comprising infectious disease specialists, clinical microbiologists, pharmacists, nurses, and administrators, should be assembled to monitor antibiotic use across departments and ensure compliance with prescribing guidelines [88,130,159,175,182-186]. Additionally, regular audits of antibiotic prescriptions can help identify patterns of overuse or misuse [187-190]. Based on audit findings, feedback can be provided to prescribers to educate them about their practices and encourage adherence to evidence-based guidelines [187-190]. Developing and implementing standardized treatment protocols for common infections, tailored to local resistance patterns, will further encourage the use of first-line antibiotics before resorting to broad-spectrum or newer drugs [88,130,159,175,182-190].

However, implementing antibiotic stewardship programs in rural areas presents challenges due to the lack of diagnostic facilities for confirming bacterial infections prior to prescribing antibiotics. Furthermore, overcrowding and understaffing in public hospitals may limit the capacity for effective stewardship implementation. It is necessary to provide essential facilities and additional staff in rural and public hospitals and initiate community-level initiatives to fully realize the benefits of antibiotic stewardship programs across India.

Community-level initiatives

Given India’s vast and diverse population, addressing ABR at the grassroots level is essential. Community-focused strategies can play a key role in this effort [8,42,107,190-192]. First, it is important to regulate over-the-counter sales of antibiotics by enforcing policies that restrict the sale of antibiotics without a prescription and strengthen pharmacy regulations to prevent indiscriminate dispensing [88,109,121,130,131,154,172,175-177,190]. Next, launching nationwide public awareness campaigns can educate people about the dangers of self-medication and the consequences of not completing antibiotic courses [42,112,116,119,122,123,125-129,132,142,157,192]. Various platforms, including mass media, social media, and grassroots outreach, can be utilized to spread this awareness in multiple languages. Engagement with local healthcare providers is also crucial [42,122,123,127,129,132,142,157,192]. Training general practitioners, traditional medicine practitioners, and pharmacists in rural and semi-urban areas on responsible antibiotic use and providing them with diagnostic tools and guidelines to distinguish between bacterial and viral infections is necessary [159,171,182-188,190]. However, cultural practices and a lack of awareness among the public often contribute to self-medication and misuse. Additionally, enforcing prescription-only policies may encounter resistance from pharmacies and local communities. 

Capacity building

Enhancing the capacity of healthcare professionals is essential for effective antibiotic stewardship, as they are pivotal in combating ABR [109,129,159,171,179,182-186,188-190]. This can be accomplished by conducting regular training programs, such as workshops and seminars, for doctors, nurses, and pharmacists on ABR and evidence-based prescribing practices [109,159,171,182-190]. It is crucial to prioritize training for healthcare workers in rural and underserved areas, where knowledge gaps are more pronounced. Integrating ABR-related topics into the curricula of medical, pharmacy, and nursing schools is also vital. Including case studies and practical exercises helps underscore the importance of antibiotic stewardship for students. Moreover, equipping hospitals and clinics with diagnostic tools, such as rapid tests to distinguish bacterial infections from viral ones, and promoting the use of microbiology laboratories for culture-based pathogen testing is essential [7,21,33,37,82,83,105,107]. However, effectively implementing these capacity-building strategies can be challenging due to limited funding and infrastructure for training programs and diagnostic facilities, particularly in rural areas [15,129,130]. Additionally, there may be resistance to change among healthcare professionals accustomed to prescribing antibiotics liberally.

Technology integration

Technology can play a transformative role in tracking antibiotic use, monitoring resistance patterns, and improving prescribing practices [193-196]. Implementing electronic health records can help monitor antibiotic prescribing patterns across hospitals and clinics [190,197,198], allowing for the analysis of collected data to identify hotspots of antibiotic misuse and resistance. Developing apps to guide healthcare providers on appropriate antibiotic use based on local resistance data is also beneficial. Offering telemedicine services can provide expert consultations in remote areas [190,199-201], thereby reducing unnecessary prescriptions. Additionally, establishing centralized databases to track antibiotic resistance trends across regions can facilitate sharing information with healthcare providers and policymakers [193-196], aiding informed decision-making. However, integrating technology faces several challenges, such as limited digital infrastructure and internet connectivity in rural areas, as well as the high costs associated with implementing and maintaining technology systems. Financial support from relevant stakeholders is necessary to realize the benefit of this strategy.

Policy and regulation

Robust policies and regulations are vital for ensuring compliance with antibiotic stewardship measures. Laws should mandate prescriptions for all antibiotic sales [121,153,154,176,177], with regular audits to monitor compliance among pharmacies and healthcare providers [159,171,182-185,188-190]. Infection prevention and control protocols should be mandated in hospitals and clinics, and antibiotic stewardship programs should be a requirement for healthcare facility accreditation [33,105,109,159,182,183,186-188,190, 200]. Consider providing financial incentives or recognition to hospitals and clinics that successfully implement stewardship programs [149]. It is also important to support pharmaceutical companies that invest in research and development related to antibiotic resistance [18,55,56,90,152,179]. However, enforcing these strategies can be challenging due to the fragmentation of India’s healthcare system, which consists of both public and private providers. Additionally, a lack of political will or funding may delay policy implementation. A collective and persistent effort by the relevant stakeholders is necessary to overcome the challenges.

Infection prevention and control (IPC)

Reducing the spread of infections is a critical part of minimizing the need for antibiotics and curbing resistance [88,130,142,143,175,202]. Ensure hospital hygiene through proper sterilization of medical equipment and adherence to hand hygiene protocols, and isolate patients with resistant infections to prevent transmission [88,130,142,143,175,203,204]. Promote vaccines to prevent infections that commonly require antibiotic treatment, such as pneumonia and typhoid, and increase vaccination coverage in rural and underserved populations [30,36,89,158,172,181]. Enhance community hygiene by improving access to clean water, sanitation, and hygiene facilities, especially in rural areas [82,142,143]. Conduct public awareness campaigns on hygiene practices, such as handwashing [42,82,110,129,132,142].

Critical appraisal of the evidence base: strengths, weaknesses, and limitations

The current review synthesizes interpretations from a broad range of sources, including government reports, surveillance databases, and published studies. While this diverse evidence base provides a comprehensive view of ABR in India, it also encompasses several limitations that need to be critically appraised. This section discusses the strengths, weaknesses, and inherent biases within the available literature, along with data gaps and potential conflicts of interest, particularly regarding industry-sponsored findings (Table 9).

**Table 9:** Critical appraisal of the evidence base

Aspect		Summary
Strengths	Diverse Data Sources	The inclusion of government reports (e.g., ICMR surveillance data) and peer-reviewed studies allow for a multidimensional perspective of the ABR crisis in India. This diversity helps highlight both national trends and localized challenges.
	Longitudinal Data	Several studies span multiple decades (from 1977 to the present), offering insights into the evolution of antibiotic resistance patterns and the impact of policy changes over time.
	Comprehensive Coverage of Mechanisms and Sectors	The review encompasses evidence regarding antibiotic mechanisms, resistance patterns in both human and animal health, and environmental impacts. This “One Health” approach is a strength that underscores the interconnectedness of various factors driving ABR.
	Policy and Regulatory Perspectives	The review draws from government policy documents and national reports, which help contextualize the epidemiological data and provide insights into the practical challenges of managing ABR in resource-limited settings.
Weaknesses	Variable Scientific Rigor	The studies and reports integrated into this review differ in methodological quality. Some sources are based on rigorous, peer-reviewed research, while others (especially certain government reports and surveillance data) may have limitations in terms of sample selection, data collection methodologies, and analytic approaches.
	Sample Bias	Many studies rely on data from tertiary care centers or urban settings, which may not be reflective of rural or community healthcare environments. This can lead to an overrepresentation of severe or healthcare-associated infections while underestimating resistance in primary care or underserved populations.
	Data Gaps	Despite the volume of available data, there remain significant gaps, especially regarding: 1) resistance patterns in rural areas and small clinics, 2) comprehensive surveillance of environmental and agricultural antibiotic use, and 3) standardized reporting of resistance among emerging pathogens. These data gaps hinder a complete understanding of the ABR scenario across all regions and settings in India.
	Heterogeneity in Data Collection and Definitions	There is inconsistency in how studies define and measure antibiotic resistance. Variations in laboratory methodology, antibiotic susceptibility breakpoints, and sample collection protocols contribute to challenges in comparing results across studies.
Risk of Bias and Potential Conflicts of Interest	Publication Bias	Studies with significant or alarming results may be more likely to be published, thereby skewing the overall picture of resistance trends. Conversely, studies with null or negative findings might be underrepresented.
	Industry Data and Conflicts of Interest	Some data included in the evidence base originate from industry-funded research or pharmaceutical companies. While these sources can offer valuable insights, they may carry an inherent risk of conflicts of interest. Industry-sponsored studies might emphasize the need for novel drug development or paint a picture that supports the commercial interests of new antibiotics. It is essential to interpret such studies with a clear understanding of their funding sources and potential biases.
	Surveillance System Limitations	Government and institutional surveillance data, although crucial, sometimes lack independent external validation. The methods used in these systems (e.g., sample collection, resistance determination) might not be standardized across regions, thereby compromising the comparability of data.

## Conclusions

ABR in India poses an urgent public health challenge that requires a coordinated, multi-pronged national response. Based on the analysis presented in this review, immediate and long-term actions must emphasize robust surveillance, enhanced antibiotic stewardship, accelerated R&D for novel treatments, and the integration of One Health strategies across sectors. To translate these priorities into concrete progress, the following action points are recommended with specific timelines, clear performance metrics, and designated responsible agencies. In the short term (within 1-2 years), the Indian Ministry of Health and Family Welfare (MoHFW), in collaboration with the Indian Council of Medical Research (ICMR), should establish a centralized, interoperable national ABR surveillance platform. This platform must integrate data from human, animal, and environmental sectors through partnerships with regional laboratories and local public health departments. A measurable metric for success will be a ≥75% geographical coverage of surveillance sites nationwide and timely reporting (monthly/quarterly) of ABR trends. Policy dashboards can be updated in real time to guide antimicrobial stewardship interventions in both urban and rural settings. Simultaneously, immediate steps should be taken to standardize and implement antibiotic stewardship programs across all healthcare facilities. The formation of a national antimicrobial stewardship task force, including representatives from both public and private sectors, should drive the rollout of standardized treatment guidelines and periodic audits. Within one year, the task force should finalize protocols and initiate training programs. Success metrics for stewardship include a target of a 30% reduction in inappropriate antibiotic prescriptions and a documented increase in compliance with best-practice guidelines, as measured by hospital audits and prescription monitoring systems.

In the medium term (2-5 years), both the MoHFW and the Department of Biotechnology (DBT) must invest in R&D to identify and develop novel antibiotics and diagnostic tools. The creation of dedicated research hubs and public-private partnerships is vital, with a goal of increasing R&D investments by at least 50% and accelerating the translation of 2-3 novel compounds or diagnostic technologies into clinical practice. Establishing clear milestones, such as the completion of pilot studies and successful clinical trials, will enable stakeholders to monitor progress and adapt strategies as needed. Finally, integrating One Health strategies requires an interministerial council that includes representatives from agriculture, veterinary sciences, environment, and public health. This council should be tasked with facilitating cross-sectoral data sharing and formulating policies that address antibiotic use in food, animals, and environmental contamination. Implementation of these policies should occur within three years, with progress monitored by metrics such as reduced antibiotic residues in environmental samples and a decrease in the incidence of resistant infections linked to animal husbandry. By establishing clear responsibilities, measurable outcomes, and defined timelines, these prioritized action points can enable India to move decisively toward mitigating ABR, ultimately leading to improved public health outcomes and a strengthened national response to antimicrobial resistance.
